# Evidence of cryptic species in the blenniid *Cirripectesalboapicalis* species complex, with zoogeographic implications for the South Pacific

**DOI:** 10.3897/zookeys.810.28887

**Published:** 2018-12-20

**Authors:** Erwan Delrieu-Trottin, Libby Liggins, Thomas Trnski, Jeffrey T. Williams, Valentina Neglia, Cristian Rapu-Edmunds, Serge Planes, Pablo Saenz-Agudelo

**Affiliations:** 1 Instituto de Ciencias Ambientales y Evolutivas, Universidad Austral de Chile, Valdivia, Chile Universidad Austral de Chile Valdivia Chile; 2 Institute of Natural and Mathematical Sciences, Massey University, Auckland, New Zealand Massey University Auckland New Zealand; 3 Auckland War Memorial Museum, Tāmaki Paenga Hira, Auckland, New Zealand Auckland War Memorial Museum Auckland New Zealand; 4 Division of Fishes, Department of Vertebrate Zoology, National Museum of Natural History, Smithsonian Institution, Suitland, MD USA National Museum of Natural History, Smithsonian Institution Suitland United States of America; 5 Mike Rapu Diving Center, Caleta Hanga Roa O’tai, Isla de Pascua, Chile Mike Rapu Diving Center Caleta Hanga Roa O’tai Chile; 6 PSL Research University: EPHE-UPVD-CNRS, USR 3278 CRIOBE, Université de Perpignan, Perpignan Cedex, France. Université de Perpignan Perpignan France; 7 Laboratoire d’Excellence “CORAIL”, Papetoai, Moorea, French Polynesia Laboratoire d’Excellence “CORAIL” Papetoai French Polynesia

**Keywords:** Austral Islands, Blenniidae, cryptic species, cytochrome oxidase I, Easter Island, endemism, French Polynesia, Gambier Islands, Kermadec Islands, mtDNA, Phylogeny, Rangitāhua, Rapa Nui

## Abstract

Rapa Nui, commonly known as Easter Island (Chile), is one of the most isolated tropical islands of the Pacific Ocean. The island location of Rapa Nui makes it the easternmost point of the geographic ranges for many western Pacific fish species that are restricted to the subtropical islands south of 20°S latitude. The blenniid fish species *Cirripectesalboapicalis* has been thought to have one of the most extensive geographic distribution ranges among these southern subtropical fish species, extending from the southern Great Barrier Reef to Rapa Nui. A phylogenetic analysis was conducted to determine the taxonomic status of the species. The results provide genetic evidence that suggests that this formerly South Pacific-wide species comprises at least three cryptic species with allopatric geographic distributions. The analyses reveal the geographic distributions of these clades and their genetic relationships with each other, and with other species within the genus *Cirripectes*. The processes that culminated in the current geographic distribution of this species complex and the zoogeographic implications of this finding for the South Pacific region are discussed.

## Introduction

The Indo-Malay-Philippines Archipelago is the hotspot of species richness for reef fishes in the Indo-Pacific region (Carpenter and Springer 2005), a richness that tends to decline with distance from this hotspot (Bellwood and Wainwright 2002; Connolly et al. 2003; Allen 2008; Briggs 2009). Accordingly, the high latitude and remote island of Rapa Nui (Easter Island, Chile), located on the eastern border of the South Pacific region, hosts one of the lowest levels of species richness reported for coral reef fishes, with only 139 shore fish species (Randall 1976; Randall and Cea 2011; Friedlander et al. 2013). The isolation of Rapa Nui has also resulted in a high proportion of endemic species (almost 22 %) (Randall and Cea 2011). The location of Rapa Nui (south of 20°S latitude) makes it the easternmost point of the geographic ranges for many subtropical Pacific fish species. These species are often either narrow-range endemics restricted to only a couple of subtropical islands of the south Pacific (e.g., *Itycirrhituswilhelmi* found only around Rapa Nui and Pitcairn Islands), or they may be widespread and occur at most of the subtropical islands south of 20°S latitude from the southern Great Barrier Reef to Rapa Nui (e.g., *Anampsesfemininus*). However, understanding the contribution of other South Pacific locations, and Rapa Nui’s own isolation, to its fish species richness and endemism is not easily answered through examination of species ranges alone. Phylogenetic analysis can provide complementary information regarding the evolutionary history of species that, together with their geographic distribution, can shed light on the origin and distribution of regional species richness.

The blenniid fish species *Cirripectesalboapicalis* (Ogilby 1899) has apparently one of the most extensive geographic distributional ranges among the southern subtropical fish species, extending from the southern Great Barrier Reef (type locality at Lord Howe Island) eastwards to Rapa Nui (Williams 1988). The taxonomic history of the Rapa Nui population of this species has not been straightforward; the first specimens collected were described as a subspecies (*Cirripectesvariolosuspatuki* (De Buen 1963)) and later elevated to the species level by Springer (1970). Williams (1988), co-author of the present study, placed the Rapa Nui endemic *C.patuki* in the synonymy of *C.alboapicalis* in his revision of the genus *Cirripectes*. The development of analytical techniques in molecular biology provides a new tool to explore taxonomic diversification and the geographic distributions of lineages at the population level and among closely-related species (Avise 2000). Given the unusually broad distribution of this subtropical species of blenny, the high level of reef fish endemism at Rapa Nui, and the taxonomic history of this species, phylogenetic analyses were conducted to evaluate the taxonomy of *C.alboapicalis* and understand the processes that shaped its geographic distribution.

## Material and methods

*Specimen collection.* Recent expeditions enabled collection of Cirripectescf.alboapicalis specimens from Rangitāhua-Kermadec Islands (LL and TT in 2015), Gambier Islands (EDT, JTW, SP in 2010), Austral Islands (EDT, JTW, SP in 2013), and Rapa Nui (EDT, VN, ECG, CRE, PSA in 2016 and 2018), while additional expeditions to the Marquesas Islands (EDT, JTW, SP) and Manuae-Scilly (JTW, SP in 2014) allowed us to collect comparative tissue samples, resulting in a total of 43 specimens of *Cirripectes* spp. for this analysis (Table 1). A variety of collecting techniques were used (Hawai’ian slings, rotenone, clove oil and hand nets). Tissues were preserved in 96% EtOH at ambient temperature.

**Table 1.** Specimens collected for this study.

Species	Geographic locality	Voucher number	GenBank number
*Cirripectes “patuki*”	Rapa Nui	RN1	MH932003
	Rapa Nui	RN2	MH932004
	Rapa Nui	RN3	MH932005
	Rapa Nui	RN4	MH932006
	Rapa Nui	RN5	MH932007
*Cirripectes* sp. n.	Austral Islands	AUST-400	MH707846
	Austral Islands	AUST-549	MH707848
	Gambier Islands	GAM-511	MH707849
	Gambier Islands	GAM-508	MH707847
	Austral Islands	AUST-550	MH707850
	Austral Islands	AUST-546	MH707855
*Cirripectes “alboapicalis*”	Kermadec Islands	Kermadecs447	MH932008
	Kermadec Islands	Kermadecs448	MH932009
* Cirripectes fuscoguttatus *	Austral Islands	AUST-157	MH707851
	Austral Islands	AUST-397	MH707852
	Austral Islands	AUST-156	MH707853
* Cirripectes jenningsi *	Austral Islands	AUST-547	MH707854
* Cirripectes quagga *	Austral Islands	AUST-165	MH707856
	Scilly Island	SCIL-193	MH707857
	Austral Islands	AUST-403	MH707859
	Austral Islands	AUST-536	MH707861
	Gambier Islands	GAM-099	MH707863
	Gambier Islands	GAM-110	MH707858
	Gambier Islands	GAM-109	MH707864
	Austral Islands	AUST-402	MH707865
	Austral Islands	AUST-537	MH707860
	Austral Islands	AUST-168	MH707862
* Cirripectes variolosus *	Austral Islands	AUST-052	MH707867
	Gambier Islands	GAM-144	MH707873
	Austral Islands	AUST-164	MH707881
	Gambier Islands	GAM-143	MH707869
	Gambier Islands	GAM-145	MH707879
	Gambier Islands	GAM-794	MH707876
	Gambier Islands	GAM-737	MH707874
	Gambier Islands	GAM-793	MH707877
	Austral Islands	AUST-162	MH707870
	Austral Islands	AUST-163	MH707880
	Scilly Island	SCIL-194	MH707875
	Scilly Island	SCIL-252	MH707866
	Austral Islands	AUST-056	MH707868
	Marquesas Islands	MARQ-071	MH707872
	Marquesas Islands	MARQ-074	MH707871
	Marquesas Islands	MARQ-073	MH707878

**Figure 1.**
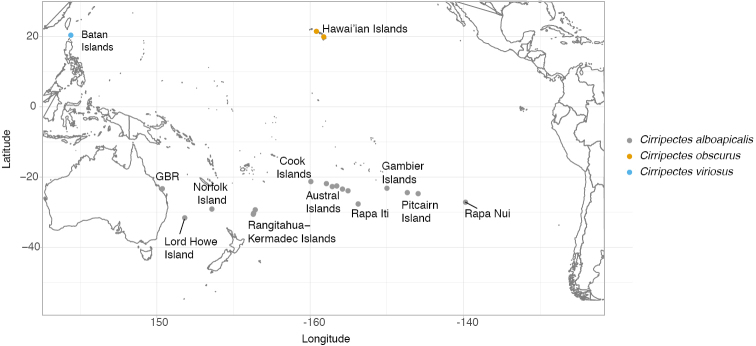
Geographic distribution of *Cirripectesalboapicalis*, *Cirripectesobscurus*, and *Cirripectesviriosus*

*Molecular analyses.* To conduct our genetic analysis, whole genomic DNA was extracted from fin clips preserved in 96% EtOH. DNA extraction was performed using GeneJet Genomic DNA purification kit (Thermo Fisher Scientific) or the DNeasy Blood & Tissue Kit (Qiagen), according to manufacturer’s protocols. A fragment of the mitochondrial gene coding for cytochrome C oxidase subunit I (COI) was amplified with the primers designed by Ward et al. (2005). PCR amplifications and sequencing were performed following the protocol of Williams et al. (2012). A 650 base-pair fragment was sequenced from each of the 43 specimens of *Cirripectes* spp. and compared with COI sequences of congeners obtained from GenBank and BOLD, with a representative of the Labrisomidae used as the outgroup (Table 1). The closest relatives of *C.alboapicalis* based on morphology are two species with very restricted distributions: *Cirripectesobscurus* (Borodin 1927), a Hawai’ian endemic species; and *Cirripectesviriosus* (Williams 1988), endemic to the Batan Islands of Philippines (northernmost islands of the Philippines) (Figure 1). We included *C.obscurus* in our study, as we collected a single specimen that was morphologically consistent with this species in the Australs; unfortunately no tissues were available from *C.viriosus* for this study. All sequences are deposited in GenBank (Table 1) and metadata uploaded to the Genomics Obervatory Metadatabase (GeOMe) (Deck et al. 2017).

Two tree-building methods were used to construct branching diagrams. First a Neighbor-joining (NJ) analysis based on the Kimura 2-parameter (K2P) model of sequence evolution (Kimura 1980) was conducted using the software package MEGA 6 (Tamura et al. 2013). Confidence in topology was evaluated by a bootstrap analysis with 1000 replicates (Felsenstein 1985). Second, a Maximum Likelihood (ML) analysis was performed using IQ-TREE (Minh et al. 2013, Nguyen et al. 2015) using the IQTREE Web Server (http://iqtree.cibiv.univie.ac.at). The best model of evolution for each partition was informed with ModelFinder (Kalyaanamoorthy et al. 2017) implemented in IQ-TREE prior to the construction of the ML tree. To assess branch support, the IQ-TREE analysis used the ultrafast bootstrap approximation (UFboot) with 1000 replicates (Minh et al. 2013) and the SH-like approximate likelihood ratio test (SH-aLRT) also with 1000 bootstrap replicates (Guindon et al. 2010). To visualize the relationships between haplotypes of *Cirripectesalboapicalis* and *C.obscurus* among the different sampling localities, a haplotype network was constructed using the haplonet function of the package “pegas” (Paradis 2010) in the R statistical environment (R Core Team 2017). Finally, estimates of Net Evolutionary Divergence (NET) between the different groups of sequences observed were computed using the software package MEGA 6 (Tamura et al. 2013) and were conducted using the K2P model (Kimura 1980).

## Results and discussion

**Figure 2.**
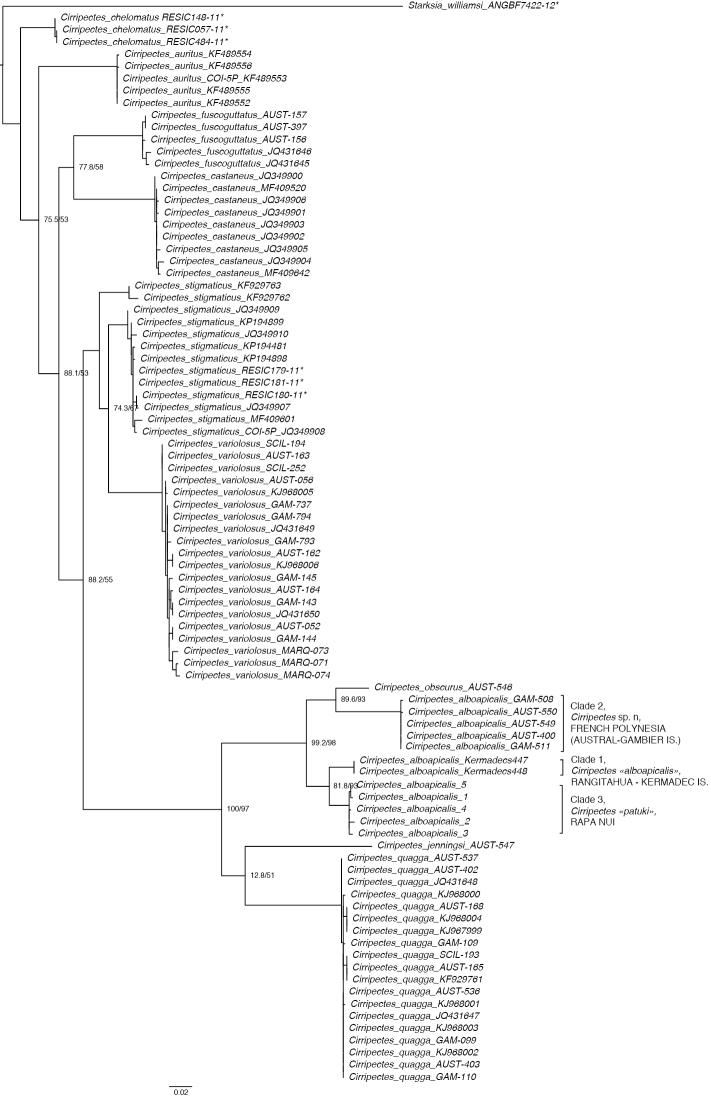
Maximum Likelihood tree for COI sequences with sequences representative of the maximum number of species retrieved from GenBank and BOLD. GenBank numbers are reported while BOLD numbers are denoted with an asterisk (*). Nodes show UFboot and SH-aLRT.

**Figure 3.**
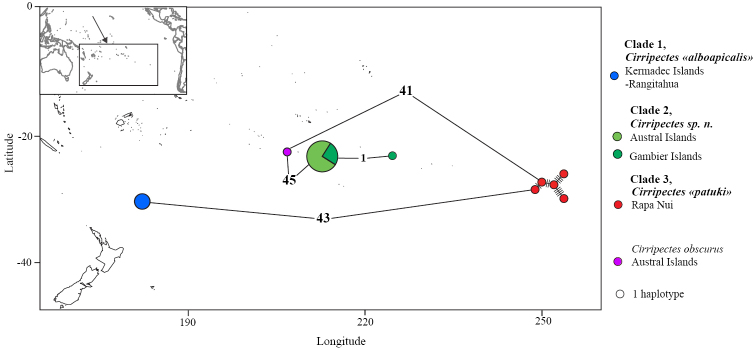
Haplotype network for the *Cirripectesalboapicalis* complex. COI sequences for *Cirripectesalboapicalis* from Austral Islands (Maria and Rurutu), Gambier Islands, Rangitāhua-Kermadec Islands (Raoul Island) and Rapa Nui. Sequence for *C.obscurus* from Austral Islands. Each circle corresponds to a unique sequence (i.e., haplotype); size of the circle indicates the frequency of the haplotype.

Molecular data were examined for 11 of the 23 valid species of the genus *Cirripectes* and included *C.obscurus*, one of the two hypothesized closest relatives of *C.alboapicalis* (based on color and morphological characters). Both the NJ and the ML analyses resulted in identical tree topologies and reveal three well-supported and highly divergent clades among the *C.alboapicalis* specimens. Clade 1 is composed of specimens from Rangitāhua-Kermadec Islands, Clade 2 of specimens from the Australs and Gambier Islands, while specimens from Rapa Nui form Clade 3 (Figure 2). The Clade 2 (Australs - Gambier) appears more closely related to the sister species *Cirripectesobscurus* than to the two other *C.alboapicalis* clades (Rangitāhua clade and Rapa Nui clade). The results from the haplotype network corroborate our phylogenetic results, as *C.alboapicalis* haplotypes form three highly divergent haplogroups. A single haplotype (from two specimens) is found in Rangitāhua and is separated by 23 mutations from a second haplogroup comprising sequences from Rapa Nui. A third haplogroup is found in the Gambier and Austral Islands and is separated by 86 mutations from the Rapa Nui haplogroup. Interestingly, the sister species, *C.obscurus*, is positioned between Clades 2 and 3 (Figure 3). Net divergence estimates ranged from 3.7 % (Clade 1–Clade 3) to 9.2 % (Clade 1–Clade 2) among the three *C.alboapicalis* clades. In contrast, net divergence between the three *C.alboapicalis* clades and *C.obscurus* ranged from 7.4 % to 7.9%. *C.alboapicalis* is thus composed of three lineages that are on different evolutionary trajectories.

**Figure 4.**
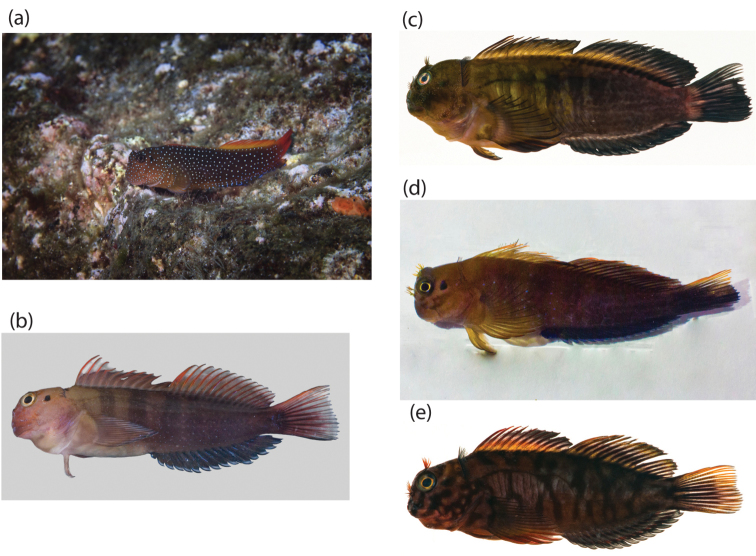
Pictures of specimens from the three genetic clades of this study; **a** live colors (photograph by Richard Robinson (www.depth.co.nz)) and **b** freshly dead colors (photograph by Carl Struthers Museum of New Zealand Te Papa Tongarewa) of Clade 1 from Rangitāhua - Kermadec Islands **c** Clade 2, French Polynesia from Austral - Gambier Islands (photographs by Jeffrey T. Williams) **d** Clade 3 Rapa Nui (photograph by Erwan Delrieu-Trottin); and **e***Cirripectesobscurus* (photograph by Jeffrey T. Williams).

Our molecular analysis reveals the existence of at least three cryptic species within the single species previously referred to as *Cirripectesalboapicalis*. In recent years, molecular studies have been combined with morphological methods and these integrated studies have led to the discovery of many new species (e.g., Baldwin et al. 2011; Delrieu-Trottin et al. 2014; and Williams and Viviani 2016). Our results provide strong justification for a detailed morphological analysis to identify diagnostic morphological characters that may distinguish the genetically divergent species within *C.alboapicalis*. Williams (1998) did not have the advantage of being able to directly compare specimens of each lineage and might easily have overlooked subtle morphological characters that might now support a morphological diagnosis of each species in addition to the genetic differentiation. A thorough morphological analysis is needed to compare the voucher specimens from each genetic lineage and to examine fresh coloration to find distinguishing characters for the three species (Figure 4).

Given that the holotype of *C.alboapicalis* is from Lord Howe Island, the species name *alboapicalis* might be retained for Clade 1 as Rangitāhua is nearest to Lord Howe Island, unless further genetic investigation suggests that Rangitāhua also harbors a distinct lineage of *C.alboapicalis*. A new name will be needed for the specimens from the Australs and Gambier Islands (Clade 2) through a formal description, while the species name *patuki* should be elevated from synonymy and attributed to the Rapa Nui population (Clade 3) provided that morphological, coloration, or other diagnostic genetic characters are found. However, such a formal species description is beyond the scope of the current study.

Results of the present study have implications for the historical zoogeography of *Cirripectes* and the historical biogeography of the region. The discovery of a specimen morphologically consistent with *C.obscurus* in the Austral Islands suggests that this species is also present in the South Pacific, outside of the Hawai’ian Islands. Although there are no publicly available COI sequences for the Hawai’ian *C.obscurus* in GenBank or BOLD, a search in the BOLD database using the identification tool (searching both public and private projects; Ratnasingham and Hebert 2007) estimated that our COI sequence for the *C.obscurus* from the Austral Islands was 99.84 % similar to sequences from three Hawai’ian *Cirripectes* larvae. Corroborating this notion that *C.obscurus* may not be a Hawai’ian endemic, but has an antitropical distribution (as defined by Hubbs (1952) and Randall (1981)), Williams (1988) also identified a potential *C.obscurus* specimen in the Cook Islands. Nonetheless, the rarity of such *C.obscurus* specimens in our collections from the South Pacific raises questions about the size of this southern population.

The full extent of the geographic distribution of the three clades identified in the blenniid *Cirripectesalboapicalis* species complex is unclear, as genetic samples from several locations across the range of this species complex are presently not available (e.g., Rapa Iti, Pitcairn Islands, Norfolk Island), and more importantly none from the type locality, Lord Howe Island. Nonetheless, the geographic distribution of the clades may follow general biogeographic patterns observed in other South Pacific species possessing a Rapa Nui population. Randall and Cea (2011) describe 17 southern subtropical fish species present in Rapa Nui including *C.alboapicalis*. Of these species, the muraenid *Gymnothoraxporphyreus* has the broadest distribution, from the southern Great Barrier Reef (GBR) to South of Chile, while an additional six species have continuous ranges between the southern GBR and Rapa Nui (Table 2). The remaining 10 species have either a very restricted distribution (e.g., *Itycirrhituswilhelmi*, *Goniistiusplessisi*, *Centropygehotumatua*) or disjunct distributions with populations in both Rangitāhua-Kermadec and Rapa Nui regions (e.g., *Aseraggodesbahamondei*, *Priolepispsygmophilia*, *Chrysipterarapanui*, see Table 2). This distribution pattern is identified as the Pitcairn-Kermadec “Province” by Rehder (1980) and includes Rapa Nui, Pitcairn, Rapa Iti, and the Rangitāhua-Kermadec Islands. Interestingly, our results suggest that the Rangitāhua-Kermadec and the Rapa Nui clades are closely related. The closest relatives of several Rapa Nui endemic species are endemic species of Rangitāhua (e.g., *Acanthistiusfuscus* and *A.cinctus*, *Girellanebulosa* and *G.fimbriata*). It is thus highly likely that both the Kermadec and the Rapa Nui clades have very restricted distributions and emerged via an allopatric process following a chance colonization.

**Table 2.** List of subtropical reef fish species that are present in Rapa Nui, and their geographic distribution (following Randall and Cea 2011). From east to west - NSW: New South Wales, S.GBR: Southern Great Barrier Reef, LH: Lord Howe Island, Nor: Norfolk Island, NC: New Caledonia, N.NZ: Northern New Zealand, R-K: Rangitāhua-Kermadec Islands, A: Austral Islands, G: Gambier Islands, Rapa: Rapa Iti, Pit.: Pitcairn, RN: Rapa Nui, JFer: Juan Fernandez, SanF: San Felix (Desventuradas Islands), Chile. Total: the total number of locations where the species is present. The three colors for *Cirripectesalboapicalis* denote the different genetic clades (see Figure 3), and grey in this row indicates the locations where the clade affinities are unknown.

Species	NSW	S. GBR	LH	Nor	NC	N. NZ	R-K	A	G	RI	Pit	RN	JFer	SanF	Chile	Total
* Cirripectes alboapicalis *	1	1	1	1			1	1	1	1	1	1				10
* Gymnothorax porphyreus *			1	1	1	1	1			1		1	1	1	1	10
* Anampses femininus *	1	1	1		1			1	1	1	1	1				9
* Bodianus unimaculatus *	1	1	1	1		1	1			1		1				8
* Enchelycore ramosa *	1		1	1		1	1			1		1				7
* Trachypoma macracanthus *	1		1	1		1	1									5
* Centropyge hotumatua *								1		1	1	1				4
* Aseraggodes bahamondei *			1	1			1					1				4
* Priolepis psygmophilia *							1			1		1				3
* Gymnothorax nasuta *										1	1	1				3
* Itycirrhitus wilhelmi *											1	1				2
* Goniistius plessisi *											1	1				2
* Chrysiptera rapanui *							1					1				2
* Bathystethus orientale *										1		1				2
